# Healthcare needs and health service utilization by Syrian refugee women in Toronto

**DOI:** 10.1186/s13031-018-0181-x

**Published:** 2018-12-03

**Authors:** Sepali Guruge, Souraya Sidani, Vathsala Illesinghe, Rania Younes, Huda Bukhari, Jason Altenberg, Meb Rashid, Suzanne Fredericks

**Affiliations:** 1 Daphne Cockwell School of Nursing, Ryerson University, POD 452A, 350 Victoria Street, Toronto, ON M5B 2K3 Canada; 2 Daphne Cockwell School of Nursing, Ryerson University, YNG 316, 415 Yonge Street, Toronto, ON M5B 2E7 Canada; 3 Daphne Cockwell School of Nursing, Ryerson University, POD 450A, 350 Victoria Street, Toronto, ON M5B 2K3 Canada; 4 Canadian Arab Institute, Suite 2500, 120 Adelaide Street West, Toronto, ON M5H 1T1 Canada; 5 Arab Community Centre of Toronto, Suite 209, 555 Burnhamthorpe Road, Toronto, ON M9C 2Y3 Canada; 6 South Riverdale Community Health Centre, 955 Queen Street East, Toronto, ON M4M 3P3 Canada; 7 Women’s College Hospital, 76 Grenville Street, Toronto, ON M5S 1B2 Canada; 8 Daphne Cockwell School of Nursing, Ryerson University, POD 474B, 350 Victoria Street, Toronto, ON M5B 2K3 Canada

## Abstract

**Objective:**

Access to healthcare is an important part of the (re)settlement process for Syrian refugees in Canada. There is growing concern about the healthcare needs of the 54,560 Syrian refugees who were admitted to Canada by May 2018, 80% of whom are women and children. We explored the healthcare needs of newcomer Syrian women, their experiences in accessing and using health services, and the factors and conditions that shape whether and how they access and utilize health services in the Greater Toronto Area (GTA).

**Method:**

This community-based qualitative descriptive interpretive study was informed by Yang & Hwang (2016) health service utilization framework. Focus group discussions were held with 58 Syrian newcomer women in the GTA. These discussions were conducted in Arabic, audio-recorded with participants’ consent, translated into English and transcribed, and analyzed using thematic analysis.

**Results:**

Participants’ health concerns included chronic, long-term conditions as well as new and emerging issues. Initial health insurance and coverage were enabling factors to access to services, while language and social disconnection were barriers. Other factors, such as beliefs about naturopathic medicine, settlement in suburban areas with limited public transportation, and lack of linguistically, culturally, and gender-appropriate services negatively affected access to and use of healthcare services.

**Conclusion:**

Responding to the healthcare needs of Syrian newcomer women in a timely and comprehensive manner requires coordinated, multi-sector initiatives that can address the financial, social, and structural barriers to their access and use of services.

## Introduction

As the conflict in Syria continues, the death toll and displacement of people in the country also continue to rise. Currently, more than 5.6 million refugees are registered with the United Nations Refugee Agency (UNHCR) and living in neighbouring countries in the Middle East and North Africa [1–4]. Further, more than 6 million people are displaced internally in Syria, and another 13.5 million people in conflict-affected areas in Syria are in need of humanitarian aid. [4, 5] As of May 2018, 54,560 Syrian refugees had been admitted into Canada, and three of four refugees are women and children. [6, 7]

Refugees to Canada may be admitted through the Government-Assisted Refugee (GAR) program, the Privately Sponsored Refugee (PSR) program, or the Blended Visa Office-Referred (BVOR) program. GAR and BVOR refugees are referred by the UNHCR or other referral agencies for (re)settlement in Canada, while PSRs are named by the respective sponsors, who are groups of Canadian citizens or coalitions (e.g., church-related organizations) [6, 8]. Although all refugee applications are screened and processed by Canadian visa officers, the referral process and the financial support provided to refugees once in Canada differ across three programs. Upon arrival to Canada, all refugees are provided with financial support for 12 months; the support is given by the federal government in the GAR program, private sponsors in the PSR program, and both parties in the BVOR program. In 2015, most Syrian refugees were admitted through the GAR program. With the federal government’s plan to increase the number of refugees accepted through the PSR program, the percentage of refugees admitted through the three programs changed in 2018 to 49% by the GAR program, 42% by the PSR program, and 9% by the BVOR program [6].

Almost half (43%) of all Syrian refugees to Canada have settled in the province of Ontario, and about 45% of them are now living in Toronto [4, 6]. Refugees admitted through the GAR program are reported to be the most vulnerable and at-risk for physical and psychological health problems [9]. All refugee claimants undergo a medical examination before they are accepted. Although applicants are not rejected because of their medical burden, they could be delayed if they have an infectious disease. The International Organization for Migration handles the arrangements for the trip to Canada, paid for by the refugee. If they cannot afford the money for the airfare, they can borrow up to $10,000 from the federal government. Once resettled, they must pay back this loan [9].

GARs receive (re)settlement assistance including temporary housing, referrals to other refugee programs, a one-time household start-up allowance, and monthly income support payment under the (Re)settlement Assistance Program (RAP). A monthly financial support is provided for each family up to one year based on social assistance rates in each province and territory. It is the minimum amount needed to cover the most basic food and shelter needs [10]. However, many find it challenging to support their families with the income support, as some must pay back the government loan for travel [11]. In some cities such as Surrey, BC, refugees are considered most at-risk for poverty, homelessness, and health problems, as a result of the stresses and struggles of (re)settlement.

After arriving in Canada, all refugees (GAR, PSR, BVOR) receive temporary coverage of health-care benefits under the Interim Federal Health Program (IFHP) until the time that they become eligible for provincial or territorial health insurance (up to 3 months, depending on the province or territory). This coverage is similar to what is available to all Canadian citizens and permanent residents under the provincial or territorial health insurance plans. It covers the costs of hospital services, visits to doctors, including visits during and after a pregnancy, and costs of tests. In addition to this basic coverage, prescription drugs and supplemental services such as, limited vision and dental care, psychotherapy, physiotherapy, and occupational and speech therapy, which are usually excluded from the provincial or territorial health coverage, are also provided for the refugees under the IFHP. The additional drug and supplemental coverage are available to refugees up to one year after (re)settlement in Canada or as long as they receive income support from the (Re)settlement Assistance Program (or equivalent in Quebec) or until the end of the private sponsorship.

Although all newcomers may experience health concerns and face barriers to accessing healthcare services in Canada, refugees face specific challenges [12]. For example, refugees from war-affected countries such as Somalia and Rwanda, and those who have been displaced numerous times, are known to be especially vulnerable to post-traumatic stress and to have had limited access to pre-migration healthcare [13, 14], and as a result, to experience higher morbidity and mortality rates than other immigrants post-migration in Canada [15].

Reports indicate that most Syrian refugees have also encountered considerable challenges prior to their entry into Canada, including war atrocities, displacement, and loss of wealth and personal materials [1]. As in all war-affected countries, women and children are particularly vulnerable [4, 16, 17]. They may have been at risk of or experienced abduction, marriage and pregnancy at an early age, sexual assault, rape, forced prostitution, and other forms of exploitation [18–22]. All of these experiences can affect the physical, psychological, and social domains of their lives.

Previous reports have documented physical health problems such as musculoskeletal pain, soft tissue injuries, disfigurement, and disabilities [18, 19]; reproductive health problems, such as sexually transmitted diseases, chronic pelvic pain, menstrual problems, and unwanted and/or high-risk pregnancies; and mental health issues [22–24].

According to some reports [22, 25, 26], Syrian women who take refuge in neighbouring countries such as Lebanon and Turkey often have limited or no access to healthcare. After arriving in Canada, the social and economic realities of adjusting to a new country and language can further affect their physical and mental health needs. Beyond what is known about the urgent health problems experienced by Syrian newcomers and the services and supports available at time of arrival, there is a lack of information about health problems that may emerge over the course of their (re)settlement, and whether and how these are being addressed. Our study explored the health needs of newcomer Syrian women in the Greater Toronto Area (GTA), their experiences in accessing health services to address these needs, and the factors that have influenced their access to and use of health services.

The study was informed by Yang and Hwang’s (2016) [27] analytical framework, which explores disparities in the utilization of health services by immigrants in relation to four categories of factors. The first category is healthcare needs, reflecting alterations in health and ongoing health concerns that prompt seeking care. The second category is resources (or lack thereof), i.e., financial and social resources that enable (or prevent) immigrants’ access to healthcare. The third category includes predisposing factors, such as individual demographic characteristics that affect access to and use of health services. The last category represents contextual conditions (e.g., socio-political, economic, healthcare) that influence access to and use of services. This framework guided the generation of questions (and probes) asked of participants during the group discussions, as well as the analysis of responses.

## Method

We used a qualitative descriptive interpretive method, which involves informed questioning and a reflective and critical examination of descriptive information to create an interpretive account and understanding of a phenomenon [28]. We conducted the study in collaboration with key health, social service, and settlement community agencies serving Syrian newcomers in the GTA, as well as with Syrian community members who had lived in Canada for some time and were knowledgeable about the community. These stakeholders were involved in the project from the beginning (the funding application stage) and provided ongoing advice and feedback about the study protocol, particularly recruitment strategies, location, and timing for data collection sessions, and the content of the focus group discussion questions. They also remain involved in knowledge uptake and dissemination activities.

Study approval was obtained from the research ethics boards at the universities and institutions of the research team (Ryerson University and Women's College Hospital). The community agencies involved in the study did not have their own ethics boards, and relied on these institutional research ethics board approvals.

Participants included Syrian newcomer women aged 18 years or older, who had been in Canada for more than 6 months but less than one year. Two recruitment strategies were used. First, community agency staff and established community members informed Syrian newcomer women they encountered about the study and provided them with contact information for the research assistant (RA). Potential participants who wanted to learn more about the study contacted the RA to obtain detailed information. Second, some participants also contacted other women in their community through word of mouth and informed them about the study.

The RA informed all participants about the study, made it clear that participation was voluntary and that participants could withdraw from the study at any time without any consequences. Before focus group sessions began, all participants provided written consent to take part in group discussions and for these to be audio-recorded.

The RA was a female graduate student who is fluent in Arabic. She collected demographic data from participants. After receiving training, she also facilitated the focus group sessions. She used open-ended questions with probes targeting the four categories of factors identified in Yang and Hwang’s (2016) [27] framework to explore health and healthcare concerns among participants (Table 1).

**Table 1:** Discussion questions

Questions Guiding Focus Group Discussions	
What type of health problems are you (and your family) experiencing?	
How do you usually deal with these problems?	
In Canada, how are you addressing these problems?	
What difficulties have you experienced in getting help for your health concerns in Canada?	
What information do you have about the healthcare system in Ontario?	
What kind of support do you need to access healthcare services in Ontario?	

Interested participants were invited to one of five focus group sessions, which were held in different locations in the GTA to maximize the diversity of groups and ensure convenience for participants. The RA informed interested participants about the date, time, and place of each focus group location (a private room at an agency serving Syrian refugees or a community centre). Interested participants arrived ahead of time to meet with the RA, review the information provided in the consent form, have any questions answered, and sign the consent form. Those who agreed to participate completed a short demographic questionnaire that captured information related to their age, marital status, language fluency, prior residence in a refugee camp, and length of stay in Canada.

In total, five focus group sessions were held in different parts of the GTA: Mississauga, North York, East York, Etobicoke, and Toronto East. The number of women attending each session ranged from 6 to 12. Each group discussion lasted 90–120 min and was audio-recorded with participants’ consent. At the end of the discussion, participants were given information about local services and supports.

Audio-recordings from the focus group sessions were transcribed and translated into English by a bilingual translator, and then verified by the RA who facilitated all focus group sessions.

Four members of the research team read the transcripts several times to gain a sense of the full dataset. They then re-read and coded one focus group transcript, paying close attention to the explicit and implicit ways in which the participants explained their health problems in relation to their pre- and post-migration context. Following this the team reached consensus on a preliminary coding scheme, which was then used to code the remaining transcripts. When new codes emerged from the data, they were compared with older codes to identify commonalities and variations, and to develop categories and subcategories that reflected the four categories of factors proposed by Yang and Hwang’s (2016) [27] framework. Data analysis ended when categories were saturated [29].

## Results

A total of 58 women, aged 21–60 years, participated in the study. At the time of data collection, they had been in Canada for 6–9 months. As shown in Table 2, most women were admitted through the GAR program, had arrived from countries neighbouring Syria, and were not living in refugee camps at the time. Most had settled in Toronto or its suburbs, and were married. All participants were fluent in Arabic; about half reported that their English fluency was good or excellent and that they were enrolled in English as an Additional Language classes.

**Table 2:** Demographic characteristics of the sample

Demographic Characteristic	Number of Participants (*n* = 58)
Immigration Stream:	
Government-Assisted Refugees (GAR)	46
Privately-Sponsored Refugees (PSR)	6
Blended Visa Office-Referred (BVOR)	4
Country of Residence Prior to Canada:	
Jordan	28
Lebanon	24
Other	6
Refugee Camp Stay Prior to Arrival in Canada:	
Yes	10
No	48
Current City of Residence:	
Toronto	31
Mississauga	12
Other	10
Current Relationship Status:	
Married	52
Non-married (widowed/separated)	6

The following results are grouped by the factors related to the four categories identified in Yang and Hwang’s (2016) [27] analytical framework: need for healthcare, resources, predisposing factors, and contextual factors.

### Need for healthcare: Pre-existing and new/emerging health needs

Participants spoke about direct experiences of war-related trauma before fleeing Syria, including detention, loss of loved ones, torture, and being exposed to chemical weapons, and how these affected their health and the health of their family. They talked about how grateful they are for the safety and security in Canada.

*I was detained and tortured in Syria. They slaughtered my husband. I am a single parent [now] and Canada has been great to me. They gave financial support for me and my daughters, and preserved my dignity. I was humiliated and tortured in my own country. Canada has been great to me and whoever says otherwise is wrong.*

*We all went through a lot; this is a new beginning for us. I thank God for not being in that place again. There are people who are still going through this. I don’t have a right to complain.*

Participants spoke about pre-existing health concerns. As listed in Table 3, they identified a range of chronic medical conditions as well as physical disabilities. For example, one participant said: *I have diabetes, and high blood pressure, and on top of it my knees hurt a lot.*

**Table 3:** Pre-existing and new/emerging health conditions

Pre-existing Conditions	New/Emerging Conditions
○ Medical conditions: asthma, infection, back pain, liver disease, kidney disease, diabetes, hypertension, psoriasis, reproductive health problems, rheumatoid arthritis, osteoporosis, sleep apnea, thyroid problems, varicose veins ○ Physical disability	○ Medical conditions: abscess, allergies, fainting, flu, fracture, vision problems, rashes, less of appetite, weight loss ○ Dental problems ○ Psychological problems: anxiety, depression, pressures and emotional exhaustion, memory loss, stress

Table 3 also lists new health concerns that emerged on their way to Canada or after arriving in Canada, including medical, dental, and psychological problems. Participants attributed their psychological problems to the distress, violence, and trauma experienced during the war, as illustrated by this quote:

*When you come [from the war], you are suffering from stress and emotional pressures and it affects you physically, and this is what is happening to me now. I have physical problems that are due to emotional stress and pressure.*

Many participants believed that their existing and/or emergent health conditions had worsened, or could potentially worsen. Many attributed these changes in their health condition(s) to delays in receipt of necessary care. They felt the response from Canadian healthcare providers was too slow to address the problems that they considered serious and/or urgent. For example, several participants were surprised by how the emergency rooms functioned:

*There is no emergency? They let people wait like that?*

*In the hospital in the emergency room, we waited ‘till 12 midnight to be seen by a doctor and all the time I was suffering from the pain.*

Participants also reported disagreement between their self-rated health and how healthcare providers evaluated their health. For example, some participants felt that healthcare providers considered their concerns about certain health problems as ‘*trivial*,’ and that the healthcare providers’ responses to their concerns were ‘*dismissive*’ and/or ‘*too slow*.’ The following excerpts capture some of these concerns:

*We have been here for six months, and I have diabetes but I have not been referred to an eye doctor. My diabetes medication is finished ... and it is a holiday, and his [the doctor’s] clinic is closed and I have no medication.*

*I have inflammation in my fingers. The doctor said “when your hand becomes swollen then we can take x-rays”. I said it feels numb but she has not done anything yet.*

*My niece fell off her bicycle, we took her to the doctor, they scheduled an x-ray after three months; an appointment in three months means they will see her when her bones are healed.*

### Resources: Financial, social and informational

Financial resources were identified as both enabling and determining factors in accessing healthcare. Participants spoke about how grateful they are for the financial support, they had in Canada. But with the end of the one-year period of government financial support and supplemental and prescription drug coverage approaching, most participants expressed concerns about future healthcare cost coverage. They were worried about the cost of medications, seeing specialists, and other healthcare services such as physiotherapy, dentists, and naturopathic and alternative medicine specialists.

Social disconnection was also a key concern for most participants. They were separated geographically from other Syrian newcomers and were trying to find ways to connect with others who could help them with the complete (re)settlement process. Most expressed the need to share resources and knowledge with people who have had similar experiences, the same struggles, and are from the same background. One participant said, ‘*I would like to hear similar stories and issues to mine.’* The majority asked how they could become more connected to and engaged in the community, how to socialize in Canada, and how to gain membership in social groups.

Limited or lack of accurate and user-friendly information about care, treatment, and services was another significant concern for participants. Throughout the focus groups, participants referred to unmet informational needs related to their healthcare access following migration to Canada. Many asked the facilitator for help in addressing their concerns. Participants explained that ‘*back home’* they used to know where to go or whom to ask about *‘who the good doctors are.’* They missed not having a similar network in Canada, especially in a new environment in which they did not even understand the language:

*Good doctors are known. When you live in your own country or neighbourhood, you get to know where to go and who to ask.*

*Where will I find this information, where to go? How to find it? It is not easily available.*

Some participants spoke about many unanswered questions, including not knowing what services were provided and by whom, and what costs are covered by health insurance. For example, one participant said:

*We don’t have the understanding on how to deal with the health system. Yesterday, I got to understand that we cannot go to the specialists directly. Maybe someone who speaks Arabic can explain [these things].*

Some participants used the Internet to access information, but many others were hindered in doing so due to lack of fluency in English, limited computer literacy, lack of access to computers and the Internet, and limited literacy overall. Most referred again to the fact that they had been accustomed to getting information from informal networks (family, friends, and neighbours) in Syria, and wanted to find similar sources in Canada. Participants identified numerous health-related information needs, which are listed in Table 4.

**Table 4:** Health-related information needs

Topic	Concerns and Information Needs
Healthcare Access	Location of specific services The role of the settlement case worker in facilitating access to healthcare Information about available emotional and mental health services and supports for themselves, husbands, and children Information about and access to naturopathic and alternative medicines Access to healthcare services for acute health problems Access to healthcare services for chronic health problems, including chronic pain management
Healthcare Coverage	Coverage of healthcare costs after the first year Healthcare resources available to government-assisted and privately sponsored refugees Services covered by Ontario Health Insurance Plan (OHIP) Services, drugs, and specialists’ services covered by various private insurance plans Information and help to understand health services offered by different service providers and settlement agencies Information about newcomers’ options following the first year of government support
Health-related Information	Coping with stress and remaining calm and relaxed Managing chronic conditions Pregnancy-related care and childbirth Accessing documents and information in Arabic

Contributing to these financial, social, and informational needs, participants identified the lack of proficient, appropriate, and timely interpretation services as a critical problem overall. Some even said that they no longer visited their doctors because the doctor did not speak Arabic:

*I had a few appointments for myself and children but I did not go because I felt it was a waste of time. We don’t understand each other. Even the person at the reception is not very helpful. She writes the appointments on the card and I cannot understand her handwriting, when I ask her to clarify, she stops me.*

*Not easy to see doctors due to the language barriers, we don’t go unless it is an emergency.*

Even the participants who spoke some English found it challenging to talk to healthcare providers:

*I can speak a little bit of English but when it comes to medical issues, I can’t express myself.*

Translators were often not available at the time of appointments; even when they were available, there was a lack of attention to the gender-specific nature of the health concerns. For example, several participants spoke about male interpreters being called in during reproductive health related appointments, and consequently not feeling comfortable discussing their concerns with the doctor.

Managing the day-to-day challenges involved in starting a life in a new country made it nearly impossible for most participants to learn English quickly and to a level of competence that allowed them to talk to healthcare providers. They also often lacked the time and financial resources to attend English classes while taking care of young children and other family members who may be experiencing mental and/or physical health problems resulting from war-affected life.

### Predisposing factors

Participants identified predisposing factors, such as their socio-cultural and immigration status, which are included in the healthcare utilization framework. Gender also played a role in determining health and healthcare needs, as reflected in how participants prioritized their family’s health. For example, all participants talked about the health concerns of their husbands and children, which they prioritized over their own in seeking and using healthcare services. This idea was captured well by one of the participants who said: ‘*we need to make sure that our family is ok, only then we can think of ourselves.’* Many participants spoke about their husbands’ mental health issues, emotional problems, and anxieties, and were concerned about how these problems may affect their future in Canada. They were also concerned about their children’s health problems, and highlighted the many health-related concerns they had about their children, from allergies to broken bones to dialysis.

Participants across focus groups expressed an interest in accessing alternative medicine in Canada. They voiced frustration about the lack of healthcare coverage for such options, as well as being laughed at and/or being discouraged openly when they asked for the option to buy alternative medicines or referrals to such specialists. One woman said:

*When I was in Jordan, I met a pharmacist, he saw my son and he asked me to use an oil on my son for his skin condition and he said you will find this in Canada. When I asked about this here, they laughed at me and they did not help me. They do not seem to respect alternative medicine.*

Syrian newcomer women’s beliefs and understandings about alternative medicine has not been explored in previous studies in Canada. However, it is commonly known that beliefs about, and the value given to, different forms of healthcare can affect healthcare-seeking behaviour [19].

### Contextual conditions

Participants tended to compare their use of health services in Canada with that of Syria and other countries that they had taken refuge in before arriving in Canada. They felt that Canadian healthcare providers’ responses to their health concerns were slow, particularly compared to what they had experienced prior to the war in Syria. As illustrated by the following excerpts, participants referred to key gaps including long wait times to see healthcare providers and delayed appointments for tests, follow-ups, and referrals:

*Everything was provided fast in Syria, you can come anytime and get yourself fixed fast.*

Most participants were accustomed to a system that allowed direct access to specialists, so they considered the inability to do this, as well as delays in referrals, to be a significant problem with the Canadian healthcare system. Some had changed family doctors because they had refused or delayed referral to a specialist:

*Back home, we go straight to the specialist.*

*I have different issues, back pain, rheumatism etc. ... my doctor knows this, he changed my medicine three or four times. It’s been three months and a half, I asked him to refer me to a specialist but he refused, saying that I do not need a specialist.*

*I have issue with my gall bladder, it has a stone. I had a crisis, they took me to the ER. They asked me to go to the family doctor so he can refer me to a specialist but the family doctor said that he cannot refer me.*

*The family doctor was supposed to refer me but it took him a long time, [so] I went to another clinic and now they have scheduled an appointment with a specialist.*

The government (re)settlement program placed immigrants in suburbs or other areas without access to public transportation, which was identified as a main barrier to healthcare utilization. Participants said that the time needed to get to appointments, and unfamiliarity with the public transit system, affected their use of healthcare services:

*The doctor is based in downtown and it is such a long commute. It is not easy, in order for me to reach the doctor’s clinic I need to take many buses.*

*I like my doctor; I take 3 buses to reach her clinic, you know how much time it takes?*

When the facilitator asked whether participants had considered finding a doctor closer to their home, one woman responded: *‘It is not easy to change a doctor. Easier said than done. Did you forget that I am a newcomer?’*

## Discussion

Previous research, both within and outside Canada, has identified some health concerns among Syrian refugees, [30, 31] and proposed guidelines for initial assessment and management of communicable and vaccine-preventable diseases in refugee-receiving countries [27, 32–36]. For example, the Canadian Collaboration for Immigrant and Refugee Health guidelines [16, 37] specify the need to focus on identifying diseases such as TB, hepatitis B, HIV, and intestinal parasitic diseases. However, most studies within and beyond Canada have not focused on newcomers’ expressed health concerns, especially beyond the immediate post-arrival period.

This is one of the first studies to examine the health concerns of Syrian newcomer women who have been in Canada for less than one year and were admitted to Canada primarily through the GAR program. Although GARs constitute only about half of the Syrian refugees admitted to Canada, they are the ones most dependent on and likely to come into contact with settlement service providers early in their (re)settlement period. GARs are more reliant on multiple and multi-levels of service providers unlike the PSRs and BVORs who have their sponsor groups’ support and help in navigating the system during the early (re)settlement period.

Like many of the refugees fleeing Syria [1], some of our participants and their families had lived in refugee camps in the countries neighbouring it, including Jordan, Lebanon, and Turkey before arriving in Canada. Some had experienced detention, torture, and loss of loved ones.

They talked about valuing the feeling of safety and security in Canada, and were grateful for the support from the Canadian government. As they shared their experiences, it became clear that they have shown great resiliency in the face of intense hardships, and continue to do so by coming forward to seek ways of improving their lives in the post-migration context in Canada.

Syrian newcomer women, like many other newcomers to Canada, have both general and immigrant-specific health needs [12]. Our participants said they had experienced delays accessing healthcare services due to lack of culturally responsive and linguistically appropriate information about the healthcare system. Notably, their healthcare service utilization appeared to be influenced by the discordance between self-reported health and how it is evaluated and responded to by healthcare providers.

Additionally, participants had certain pre-arrival perceptions and expectations about Canada’s healthcare system, which were not met by their actual experiences with the system, their healthcare providers, and the nature of healthcare services provided and received. They were surprised by the limited information available about mental health services, dental care, eye care, specialized services for children, as well as services related to reproductive health, pregnancy, and childbirth.

Some of these concerns may be related to poor communication on the part of healthcare providers in explaining their rationale for time taken for referrals and diagnostic tests. For example, if HbA1c is ‘normal’ (indicative of blood sugar under control long term) and if there are no other related concerns, an eye examination might not be a priority in relation to other health concerns; and medication reordering may have been done through a pharmacy.

They also referred to information gaps at the broader systems-level with regard to access, funding, referral, and follow-up.

Predisposing factors such as gender, immigration status, pre-migration experiences, and length of stay in Canada appear to intersect with contextual conditions to shape health service utilization experiences.

Among our participants, access to healthcare was shaped by the available resources. Receiving health insurance encouraged health service utilization, while social disconnectedness and the lack of appropriate and adequate language resources, in particular, prevented it. Participants identified isolation, language barriers, not being connected with Arabic-speaking healthcare providers, lack of serious attention to their health concerns, and slow follow-up and referrals as key reasons for their declining health status.

With the end of the one-year period of government financial support and the supplemental and prescription drug benefits coverage period approaching, many participants had fears about the future, specifically finding work, learning English, supporting their families, and accessing health, social, and settlement services.

Informal networks of friends, families, and ethnic and religious communities are known to bridge language barriers, mitigate mistrust of the system, and also act as a buffer against the harmful effects of stress during (re)settlement [38, 39]. A similar protective network has not be available to Syrian newcomers, given the rapid (re)settlement process that dispersed families across the different parts of the GTA and beyond, creating a considerable disconnect from each other. Many of the advocacy groups, non-profit groups, and settlement services that responded to the initial call to action for receiving Syrian refugees into Canada have not had the financial or social resources needed to continue their work beyond this initial period. Provincial-level refugee-specific resources and networks may be unable to provide support to refugees and their integration into the healthcare system beyond the initial (re)settlement period [4].

## Conclusion

Together, the results of this study point to the need for a broader understanding of refugee health needs, beyond a simple examination of pre-migration risks or a focus on the cultural determinants of healthcare seeking behaviour. Beyond efforts to address the immediate health needs of Syrian refugees, it is crucial to develop a multisector coordinated approach that takes the many layers of health needs, resources/barriers, predisposing factors, and social and structural conditions into account. Our study demonstrated the usefulness of an analytical framework for not only understanding healthcare access experiences and mapping the different elements of it, but also exploring the way in which predisposing factors such as gender, immigration status, pre-migration experiences, appear to intersect with contextual conditions to shape health service utilization experiences. Similar, theoretically informed studies could be useful in better understanding the (re)settlement experiences and healthcare utilization among not only Syrian refugees, but other newcomers and refugees across a larger timespan, in other areas in Canada. This will contribute to an evidence base in support of a broader inclusivity/diversity-based approach to newcomer (re)settlement policymaking in Canada.
